# IL-15 enhances HIV-1 infection by promoting survival and proliferation of CCR5^+^CD4^+^ T cells

**DOI:** 10.1172/jci.insight.166292

**Published:** 2023-04-10

**Authors:** Yuhao Li, Hongbo Gao, Kolin M. Clark, Liang Shan

**Affiliations:** 1 Division of Infectious Diseases, Department of Medicine, and; 2 The Andrew M. and Jane M. Bursky Center for Human Immunology and Immunotherapy Programs, Washington University School of Medicine, St. Louis, Missouri, USA.

**Keywords:** AIDS/HIV, Immunology, Cytokines, T cells

## Abstract

HIV-1 usually utilizes CCR5 as its coreceptor and rarely switches to a CXCR4-tropic virus until the late stage of infection. CCR5^+^CD4^+^ T cells are the major virus-producing cells in viremic individuals as well as SIV-infected nonhuman primates. The differentiation of CCR5^+^CD4^+^ T cells is associated with the availability of IL-15, which increases during acute HIV-1 infection. Here, we report that CCR5 was expressed by CD4^+^ T cells exhibiting effector or effector memory phenotypes with high expression levels of the IL-2/IL-15 receptor common β and γ chains. IL-15, but not IL-7, improved the survival of CCR5^+^CD4^+^ T cells, drove their expansion, and facilitated HIV-1 infection in vitro and in humanized mice. Our study suggests that IL-15 plays confounding roles in HIV-1 infection, and future studies on the IL-15–based boosting of anti–HIV-1 immunity should carefully examine the potential effects on the expansion of HIV-1 reservoirs in CCR5^+^CD4^+^ T cells.

## Introduction

CCR5 and CXCR4 are 2 coreceptors for HIV-1 entry (1, 2). CCR5-tropic viruses almost always dominate the pool of viruses in blood and tissues early after acquisition, regardless of the route of viral transmission (2–6). CXCR4-tropic viruses emerge during later stages of infection in approximately 50% of people living with HIV (PLWH) that do not take antiretroviral therapy (ART) (7). CCR5 is expressed on a fraction of activated CD4^+^ T cells and some effector memory CD4^+^ T cells, which are not truly resting, often exhibit an exhaustive phenotype, and are prone to cell death (8–11). By contrast, naive and central memory CD4^+^ T cells rarely express CCR5 and may switch on CCR5 expression via activation and proliferation (12). In nonhuman primates (NHPs), the host cells that support SIV replication include activated and recently activated (ostensibly resting) effector memory CD4^+^ T cells that contain high concentrations of CCR5 mRNA (13–15). In viremic individuals, CCR5^+^CD4^+^ T cells with high levels of activation and exhaustion marker expression (CD38^+^HLADR^+^) are the predominant population of cells containing HIV-1 RNA (16). CCR5^+^CD4^+^ T cells are enriched in mucosa-associated lymphoid tissue and are rare in blood and secondary lymphoid organs (17) because CCR5 regulates recruitment of immune cells to sites of inflammation (18). In addition, these cells either actively produce type 1 and type 17 cytokines or have cytolytic capacities (19–23) and are often at transitioning stages, either dying quickly or differentiating into quiescent memory cells (12, 24, 25).

Most studies utilize HIV-1 reporter viruses pseudotyped with VSV-G or CXCR4-tropic envelopes for bulk CD4^+^ T cells, which do not precisely model the fate of CCR5^+^CD4^+^ T cells upon viral infection. However, these cells are the predominant cellular targets for HIV-1, produce high levels of HIV-1 RNA, and are essential to the seeding of latent viral reservoirs (24). Previous studies implicated IL-15 in expansion of CCR5^+^CD4^+^ T cells (25, 26), and plasma IL-15 concentration has a positive correlation with plasma HIV-1 or SIV RNA levels (27, 28). Administration of IL-15 in rhesus macaques (RMs) selectively expanded CCR5^+^CD4^+^ T cells that accumulated in extralymphoid tissues (29). In addition, depletion of CD8^+^ T cells in RMs resulted in rapid expansion of CCR5^+^CD4^+^ T cells, which was completely inhibited by IL-15 neutralization (30), suggesting that CD8^+^ and CD4^+^ T cells compete for IL-15. Although the effects of IL-15 on memory CD4^+^ T cells has been studied extensively, the cellular mechanisms governing IL-15–mediated regulation of HIV-1 target cells remain unclear. In the present study, we aimed to understand the role of IL-15 in the survival and proliferation CCR5^+^CD4^+^ T cells prior to and during HIV-1 infection.

## Results

### IL-15 promotes survival of CCR5^+^CD4^+^ T cells.

**Figure 1:**
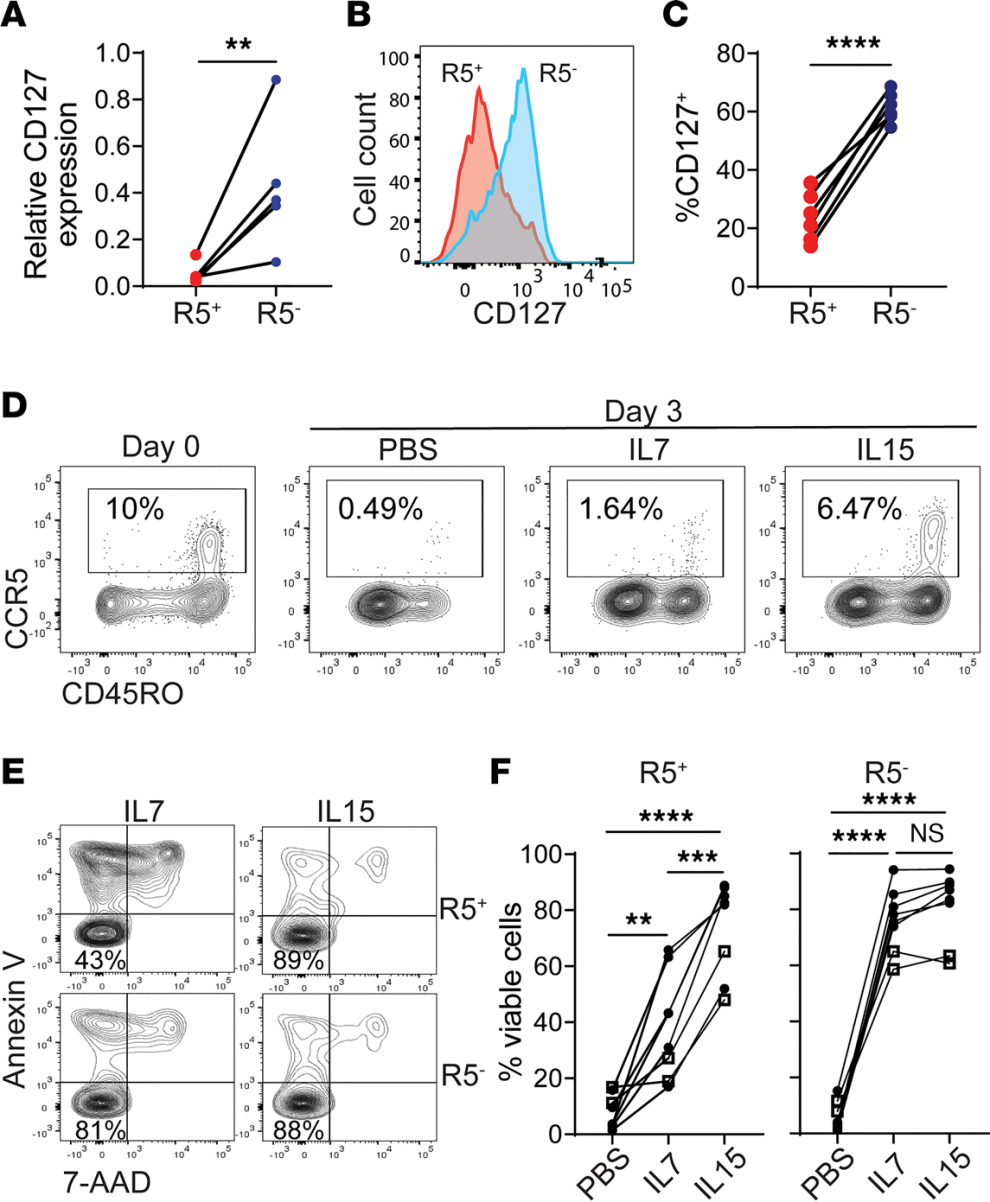
IL-15 enhances survival of CCR5^+^CD4^+^ T cells. (**A**–**C**) CD127 expression in unstimulated CCR5^+^ and CCR5^–^ tonsillar CD4^+^ T cells. (**A**) CCR5^+^ and CCR5^–^ cells were purified by sorting for RNA extraction and cDNA synthesis. *CD127* expression levels were normalized to *POLR2A*. *n* = 5. (**B** and **C**) CD127 expression was determined by flow cytometry. *n* = 6. (**D**) Freshly isolated tonsillar CD4^+^ T cells were cultured in the presence of IL-7 or IL-15 for 3 days. Frequency of remaining CCR5^+^ cells was determined by flow cytometry. (**E** and **F**) Viability of CCR5^+^ or CCR5^–^ CD4^+^ T cells. Blood and tonsillar CCR5^+^ and CCR5^–^ cells were purified by sorting and then cultured in the presence of PBS, IL-7, or IL-15 for 3 days. Solid circles indicate blood (*n* = 6). Hollow squares indicate tonsils (*n* = 2). *P* values were calculated using a paired, 2-tailed *t* test (**A** and **C**) or 1-way ANOVA with Tukey’s multiple-comparison test (**F**). **P < 0.01; ***P < 0.001; ****P < 0.0001.

CCR5 is rarely expressed by quiescent CD4^+^ T cells and is upregulated following T cell activation (24). The common γ chain cytokine family members IL-2, IL-7, and IL-15 exert a major influence on the survival of expanding T cell clones (31). IL-2 is produced almost exclusively by activated T cells and regulates T cell proliferation and survival in an autocrine manner (32). Minimal IL-2 production can be detected in T cells undergoing the effector-to-memory transition. By contrast, IL-7 and IL-15 are produced by stromal cells and other immune cells, including monocytes, macrophages, and dendritic cells to regulate memory T cell survival (31). IL-7 and IL-15 play major roles in facilitating the transition of memory precursor T cells to quiescent memory cells. The IL-7 receptor complex includes the IL-2 common γ chain (IL-2RG, or CD132) and the IL-7 receptor α subunit (IL-7RA, or CD127), which is usually downregulated in activated CD4^+^ T cells. We first determined which cytokine(s) could promote the survival of CCR5^+^ effector CD4^+^ T cells. We analyzed CD127 expression in freshly isolated tonsillar CD4^+^ T cells and found that the CCR5^+^ cells had significantly lower transcript levels (Figure 1A) and surface expression of CD127 (Figure 1, B and C). When tonsillar CD4^+^ T cells were cultured in the presence of IL-7, the frequency of CCR5^+^ cells decreased rapidly, while the loss of CCR5^+^ cells was partially prevented by IL-15 (Figure 1D). To determine whether the loss of CCR5^+^ cells was due to cell death or CCR5 downregulation, we purified CCR5^+^ and CCR5^–^ cells to measure cell viability. We found that CCR5^+^CD4^+^ T cells respond preferentially to IL-15 to maintain their survival, while CCR5^–^CD4^+^ T cells were able to utilize both IL-7 and IL-15 (Figure 1, E and F).

### IL-15 promotes proliferation of CCR5^+^CD4^+^ T cells.

**Figure 2:**
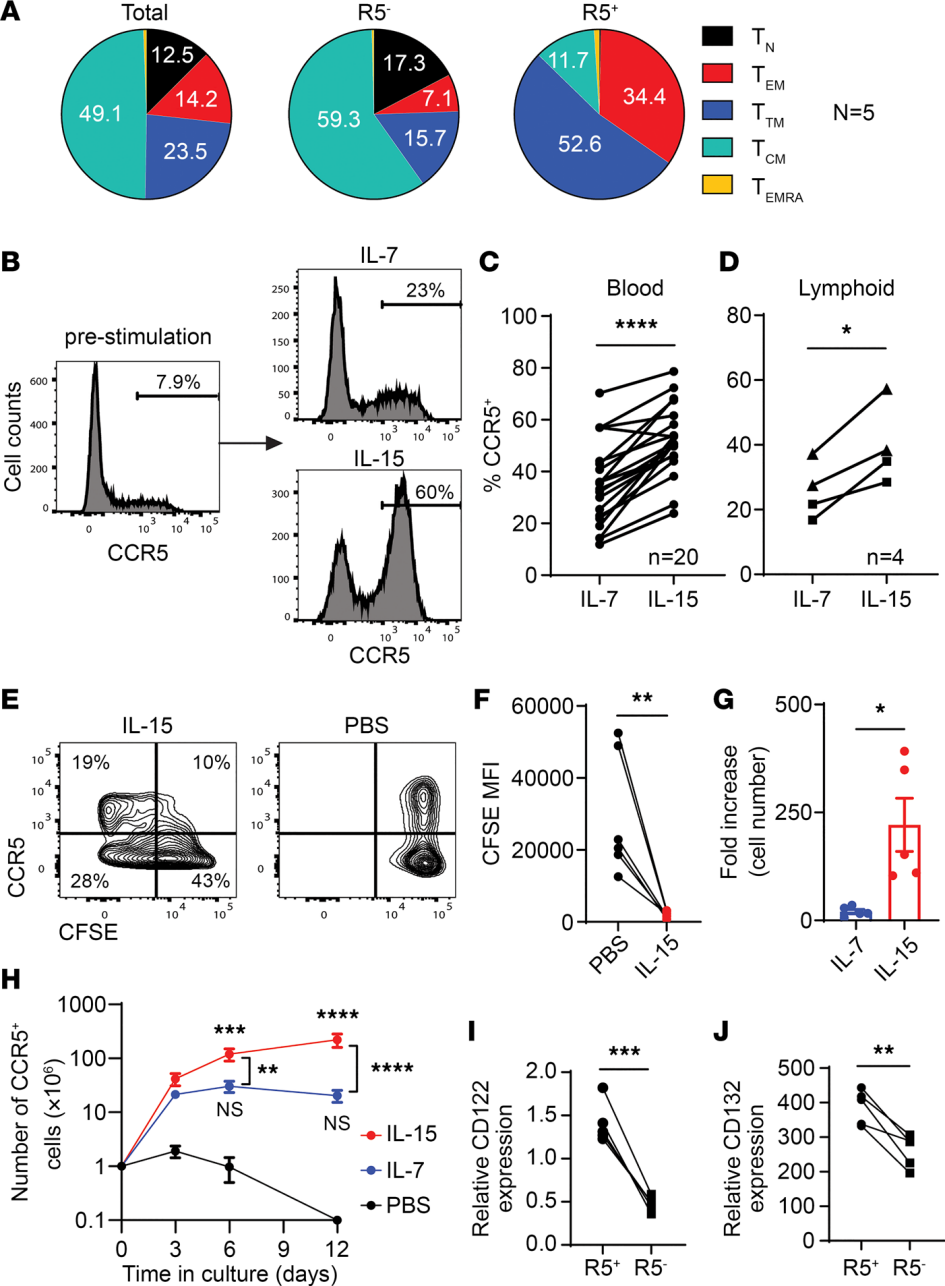
IL-15 drives proliferation of CCR5^+^CD4^+^ T cells. (**A**) The distribution of memory T cell subsets among total, CCR5^–^, and CCR5^+^ CD4^+^ T cells. Blood samples were collected from 5 heathy donors without stimulation. Tn, naive T cells (CD45RA^+^CCR7^+^); Tem, effector memory T cells (CD45RA^–^CCR7^–^CD27^–^); Ttm, transitional memory T cells (CD45RA^–^CCR7^–^CD27^+^); Tcm, central memory T cells (CD45RA^–^CCR7^+^CD27^+^); Temra, RA^+^ effector memory T cells (CD45RA^+^CCR7^–^). (**B**–**D**) Differentiation of CCR5^+^CD4^+^ T cells following T cell activation. CD4^+^ T cells from blood, tonsils, or lymph nodes were costimulated with anti-CD3 and anti-CD28 antibodies for 3 days and then cultured in the presence of IL-7 or IL-15 for 6 days. Frequency of CCR5^+^ cells was determined by flow cytometry. Circles indicate blood (*n* = 20), squares indicate tonsils (*n* = 2), and triangles lymph nodes (*n* = 2). (**E**–**H**) Expansion of CCR5^+^CD4^+^ T cells by IL-15. Total CD4^+^ cells from blood were costimulated with anti-CD3 and anti-CD28 antibodies for 3 days. Activated cells were then stained with CFSE and cultured in the presence of IL-7 or IL-15. Mean CFSE intensity and number of CCR5^+^ cells were determined by flow cytometry. In **F**, CD4^+^ T cells from 6 blood donors were included. In **G**, fold increase was calculated on day 12. CD4^+^ T cells from 5 blood donors were included. (**I** and **J**) *CD122* and *CD132* expression in CCR5^+^ and CCR5^–^ CD4^+^ T cells. CCR5^+^ and CCR5^–^ cells were purified by sorting for RNA extraction and cDNA synthesis. *CD122* and *CD132* expression levels were normalized to *POLR2A*. CD4^+^ T cells from 5 blood donors were included. *P* values were calculated using a paired, 2-tailed *t* test (**C**, **D**, **F**, **G**, **I**, and **J**) or 2-way ANOVA with Tukey’s multiple-comparison test (**H**). *P < 0.05; **P < 0.01; ***P < 0.001; ****P < 0.0001.

Resting CD4^+^ T cells — including naive and central memory cells — typically are CCR5^–^, some of which may turn on CCR5 expression after activation. By contrast, the vast majority of CCR5^+^CD4^+^ cells are effector or transition memory cells (Figure 2A and Supplemental Figure 1; supplemental material available online with this article; https://doi.org/10.1172/jci.insight.166292DS1), which are more active and more differentiated than their CCR5^–^ counterparts, suggesting that most CCR5^+^CD4^+^ T cells are recently activated cells. To recapitulate this process, blood, tonsillar, or lymph node CD4^+^ T cells were stimulated with anti-CD3 and anti-CD28 antibodies for 3 days and then cultured in the presence of IL-7 or IL-15 for another 6 days (Figure 2B). As expected, a higher frequency of CCR5-expressing cells was observed when activated blood and lymphoid CD4^+^ T cells were cultured in the presence of IL-15 (Figure 2, B–D). We hypothesized that increased cell viability might not be the only reason that IL-15 treatment led to a higher frequency of CCR5^+^ cells. We then determined whether IL-15 could drive proliferation of these cells. The proportion of CCR5^+^CD4^+^ T cells that proliferated under IL-15 stimulation and became CFSE^lo^ was higher than CCR5^–^ cells, and IL-15 reduced the CFSE mean fluorescence intensity of CCR5^+^CD4^+^ T cells by 15-fold compared with nonproliferating controls (Figure 2, E and F). IL-15 treatment led to a 200-fold increase in the numbers of CCR5^+^CD4^+^ T cells (Figure 2, G and H). Although CD4^+^ T cells do not express IL-15 receptor α chain (IL-15RA, or CD215) (Supplemental Figure 2A), we found that CCR5^+^CD4^+^ T cells expressed high levels of IL-2 common β chain (IL-2RB, or CD122) and CD132 (Figure 2, I and J, and Supplemental Figure 2), which is likely one of the reasons that CCR5^+^ cells respond to IL-15 more efficiently than their CCR5^–^ counterparts.

**Figure 3:**
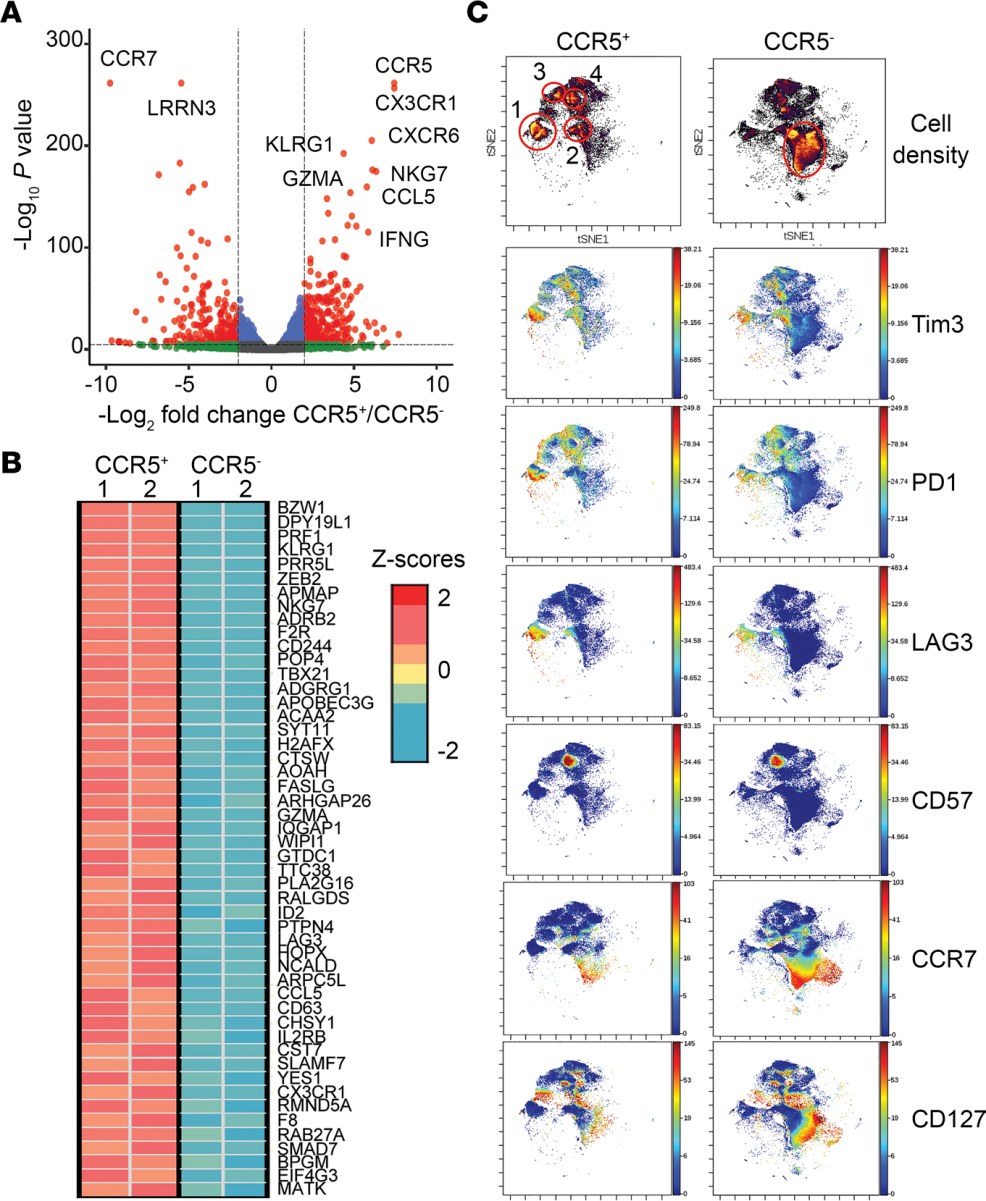
Transcriptional and phenotypic profiling of CCR5^+^CD4^+^ T cells. (**A**–**C**) Activated blood CD4^+^ T cells were cultured in the presence of IL-15 for 6 days. CCR5^+^ and CCR5^–^ cells were purified by sorting for RNA-Seq (**A** and **B**) or mass cytometry (**C**). (**A**) Volcano plot of 1,602 genes differentially expressed in CCR5^+^ and CCR5^–^ cells. (**B**) Heatmap of genes associated with T cell activation and their relative expression levels in CCR5^+^ and CCR5^–^ cells. (**C**) viSNE plots show cell density or expression levels of indicated genes. CD4^+^ T cells from 2 blood donors were included.

Next, bulk RNA sequencing (RNA-Seq) was performed to compare between CCR5^+^ and CCR5^–^ CD4^+^ T cells and the data were analyzed for differential expression using DESeq2, resulting in 1,602 differentially expressed genes at a conservative *P*-value cutoff of 1 × 10^–6^. Some of the most significantly differentially expressed genes can be seen in Figure 3A, such as *KLRG1*, *CCR5*, and *IFNG* for CCR5^+^ cells and *CCR7* for CCR5^–^ cells. To further assess the gene signature differences between these 2 populations, we conducted gene set enrichment analysis (GSEA) using MSigDB’s GSEA software (v2.3) and the immunologic gene signatures set to identify particular immunologic profile differences between CCR5^+^ and CCR5^–^ cells. One of the most significant differentially expressed gene sets was the unstimulated versus activated CD4^+^ T cells (enrichment score of 0.474 and FWER *P* value of 0). This indicates that CCR5^+^CD4^+^ T cells displayed a transcriptional signature that was more active, whereas CCR5^–^ cells displayed a more resting profile. A heatmap of the top genes expressed in CCR5^+^ cells from this gene set is shown in Figure 3B. Next, we performed cytometry by time of flight (CyTOF) to understand IL-15–mediated expansion of CCR5^+^ cells and identified 4 CCR5^+^ clusters (Figure 3C and Supplemental Figure 3). None of these expanded clusters expressed CD127 or CCR7. Cluster 1 was positive for Tim3, PD-1, and LAG3, while clusters 2 and 3 were positive for Tim3/LAG3 and Tim3/PD-1, respectively. Cluster 4 contained CD57-expressing cells, which marks late-stage differentiated or senescent lymphocytes. By contrast, treatment of CCR5^–^CD4^+^ T cells with IL-15 led to expansion of resting central memory cells expressing CCR7 and CD127 but not activation or exhaustion markers. We also performed cytometry analysis and confirmed the emergence of the CD57^+^ population, most of which were CCR5^+^ cells (Supplemental Figure 4A) and had lower CFSE intensity than CD57^–^CCR5^+^ cells (Supplemental Figure 4, B and C), suggesting that this subset of cells underwent more rounds of proliferation than other CCR5^+^ cells.

### IL-15 promotes CCR5-tropic HIV-1 infection.

**Figure 4:**
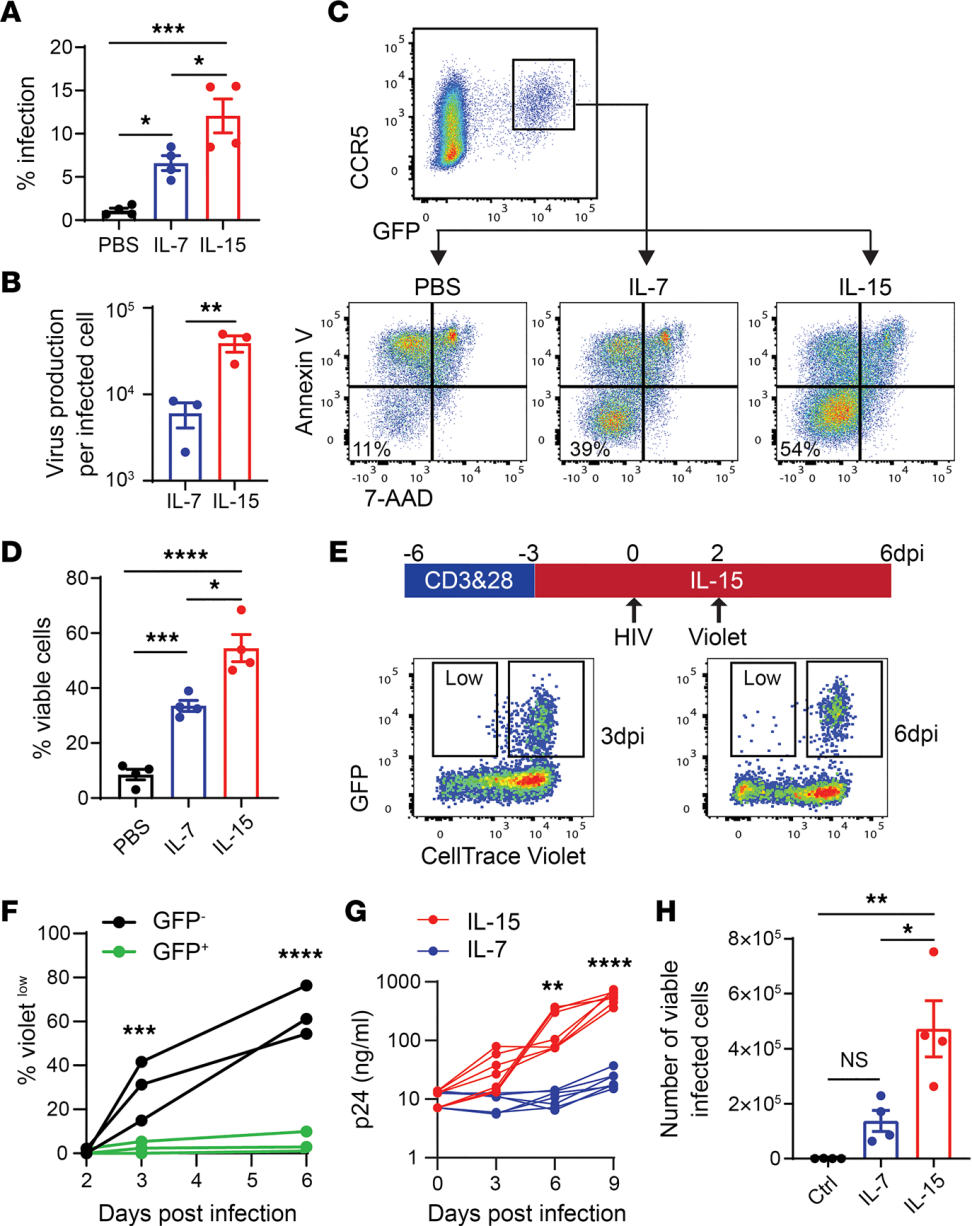
IL-15 promotes replication of CCR5-tropic HIV-1. (**A** and **B**) Activated blood CD4^+^ T cells were cultured in the presence of IL-7 or IL-15 for 6 days. Cells were infected with the single-round HIV-1 reporter virus NL4-3Δenv-EGFP pseudotyped with the Yu2 envelope. (**A**) GFP expression was measured 3 days post infection (dpi). *n* = 4. (**B**) Virus production per infected cell was determined by quantification of copies of HIV-1 RNA in supernatant, divided by the number of GFP^+^ cells in the culture. *n* = 3. (**C** and **D**) Survival of HIV-1–infected cells. Infection was performed as described in **A**. GFP^+^ cells were purified by cell sorting on 3 dpi and were cultured with IL-7 or IL-15 for 3 days. Cell viability was determined by flow cytometry. CD4^+^ T cells from 4 blood donors were included in this experiment. (**E** and **F**) Proliferation of HIV-1–infected cells. Infection was performed as described in **A**. Cells were stained with CellTrace Violet on 2 dpi. Proliferation of infected and uninfected cells was measured on 3 and 6 dpi. CD4^+^ T cells from 3 blood donors were included. (**G** and **H**) Quantification of HIV-1 replication. Activated blood CD4^+^ T cells were infected with HIV_Ba-L_ for 9 days in the presence of IL-7 or IL-15. (**G**) Culture supernatant was collected on day 3, 6, and 9 for p24 ELISA. CD4^+^ T cells from 10 blood donors were included. (**H**) Viable infected cells on day 9 were determined by flow cytometry. CD4^+^ T cells from four blood donors were included. *P* values were calculated using 1-way ANOVA with Tukey’s multiple-comparison test (**A**, **D**, and **H**), paired, 2-tailed *t* test (**B**), or 2-way ANOVA with Holm-Šidák multiple-comparison test (**F** and **G**). *P < 0.05; **P < 0.01; ***P < 0.001; ****P < 0.0001.

Since IL-15 promotes survival and proliferation of CCR5^+^CD4^+^ T cells, it should facilitate replication of CCR5-tropic HIV-1 by increasing the availability of target cells. In addition, IL-15 induces SAM domain– and HD domain–containing protein 1 (SAMHD1) phosphorylation, relieving the inhibition of reverse transcription (33). As expected, CD4^+^ T cells pretreated with IL-15 had a higher percentage of infection by a CCR5-tropic HIV-1 reporter virus (Figure 4A). We found that the virus burst size was nearly 10-fold larger in IL-15–treated cells (Figure 4B), likely because cells were more active when treated with IL-15. Next, we asked whether IL-15 could promote survival and proliferation of HIV-1–infected CCR5^+^CD4^+^ T cells. We purified cells infected by a CCR5-tropic HIV-1 reporter virus (GFP^+^), which were exclusively CCR5^+^. These cells were cultured for 3 days before cell viability and viral RNA measurement (Figure 4C). Both IL-7 and IL-15 increased the viability of infected cells and IL-15 was more effective than IL-7 (Figure 4D). To monitor cell proliferation, CCR5^+^CD4^+^ T cells were infected by a CCR5-tropic HIV-1 reporter virus and then labeled with CellTrace Violet dye. Although uninfected cells proliferated quickly under IL-15 treatment, the infected cells remained Violet^hi^ (Figure 4, E and F), suggesting that IL-15 could not overcome the cell cycle arrest caused by HIV-1 proteins such as Vpr and Vif (34). To evaluate viral replication, we used the replication-competent R5-tropic virus HIV_Ba-L_ to infect CD4^+^ T cells treated with IL-7 or IL-15. Both the number of viable infected cells (Annexin V^–^7-AAD^–^p24^+^) and the production of viral particles were significantly increased by IL-15 (Figure 4, G and H). Notably, we only monitored the survival and proliferation of productively infected cells (GFP^+^ or p24^+^). It is possible that the infected CCR5^+^CD4^+^ T cells would have begun to proliferate in response to IL-15 as soon as the virus entered the latent state.

### IL-15 promotes HIV-1 infection in humanized mice.

**Figure 5:**
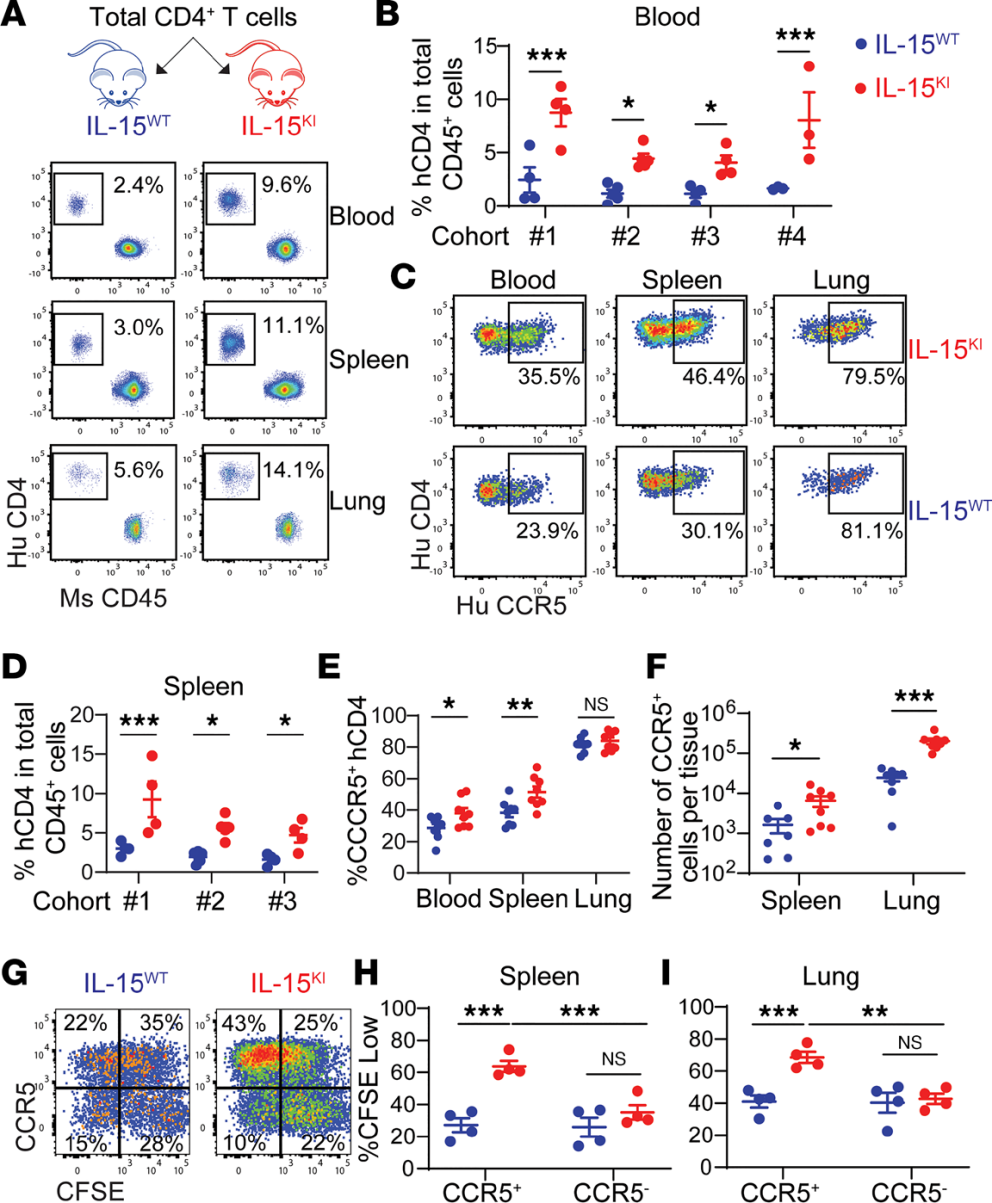
Differentiation and proliferation of CCR5^+^CD4^+^ T cells in humanized mice. (**A**–**F**) Total blood CD4^+^ cells from 4 different donors were costimulated with anti-CD3 and anti-CD28 antibodies for 3 days before transfused into IL15^WT^ and IL15^KI^ mice by retro-orbital injection. Each mouse received 5 × 10^6^ cells. Blood and tissues were collected on day 6 after transfusion. The percentage of human CD4^+^ T cells of total CD45^+^ cells in blood (**A** and **B**) or spleen (**D**) from IL15^WT^ and IL15^KI^ mice was determined by flow cytometry. The frequency (**C** and **E**) and the number (**F**) of CCR5^+^CD4^+^ T cells in blood, spleens, and lungs are shown. In **E** and **F**, data were from cohort 1 and 2 combined. Group sizes in cohorts 1–4 are 4, 5, 4, and 3, respectively. (**G**–**I**) Activated CD4^+^ T cells were labeled with CellTrace CFSE before being transfused into IL15^WT^ and IL15^KI^ mice by retro-orbital injection. Tissues were collected on day 9 after transfusion. (**G**) Representative plots of cells from spleens. (**H** and **I**) Proportion of proliferated (CSFE^lo^) CCR5^+^ and CCR5^–^ cells from spleen and lung. Each group contains 4 mice. *P* values were calculated using 2-way ANOVA with Holm-Šidák multiple-comparison test. *P < 0.05; **P < 0.01; ***P < 0.001.

Delivering human IL-15 into humanized mouse systems is often used to improve human NK cell and T cell responses due to the poor cross-reactivity between murine IL-15 and the human receptors. However, the delivery approaches that include IL-15 protein injection and transgenic or viral vector–mediated IL-15 expression are not physiologically relevant because the serum IL-15 concentration in these models (1–100 ng/mL) is orders of magnitude above normal concentrations (<10 pg/mL). To understand the roles of IL-15 in the differentiation of CCR5^+^CD4^+^ T cells and HIV-1 infection in vivo, we generated human IL-15–knockin (IL15^KI^) mice as previously described (35). The IL-15 concentration in IL15^KI^ mice was 60–70 pg/mL (Supplemental Figure 5), which is 5- to 10-fold higher than its physiological concentration in humans, but is much lower than other mouse models. First, we transfused bulk blood CD4^+^ T cells into the IL15^KI^ or control (IL15^WT^) mice and found that the frequency of human CD4^+^ T cells in blood was significantly higher in IL15^KI^ mice (Figure 5, A and B), mainly due to the increased frequency and number of CCR5^+^CD4^+^ T cells (Figure 5, C–F). To determine the proliferation of CCR5^+^CD4^+^ T cells, CFSE-labeled blood CD4^+^ T cells were transfused into the 2 groups of mice. CCR5^–^CD4^+^ T cells had comparably low proliferation capacity in both IL15^KI^ and IL15^WT^ mice, whereas CCR5^+^CD4^+^ T cells expanded more efficiently in IL15^KI^ mice, most of which became CFSE^lo^ (Figure 5, G–I). CCR5^+^CD4^+^ T cells that express CD57 also emerged in IL15^KI^ but not IL15^WT^ mice (Supplemental Figure 6), likely due to hyperproliferation of these cells in the presence of IL-15. These results suggest that IL-15 facilitated differentiation and expansion of CCR5^+^CD4^+^ T cells in vivo.

**Figure 6:**
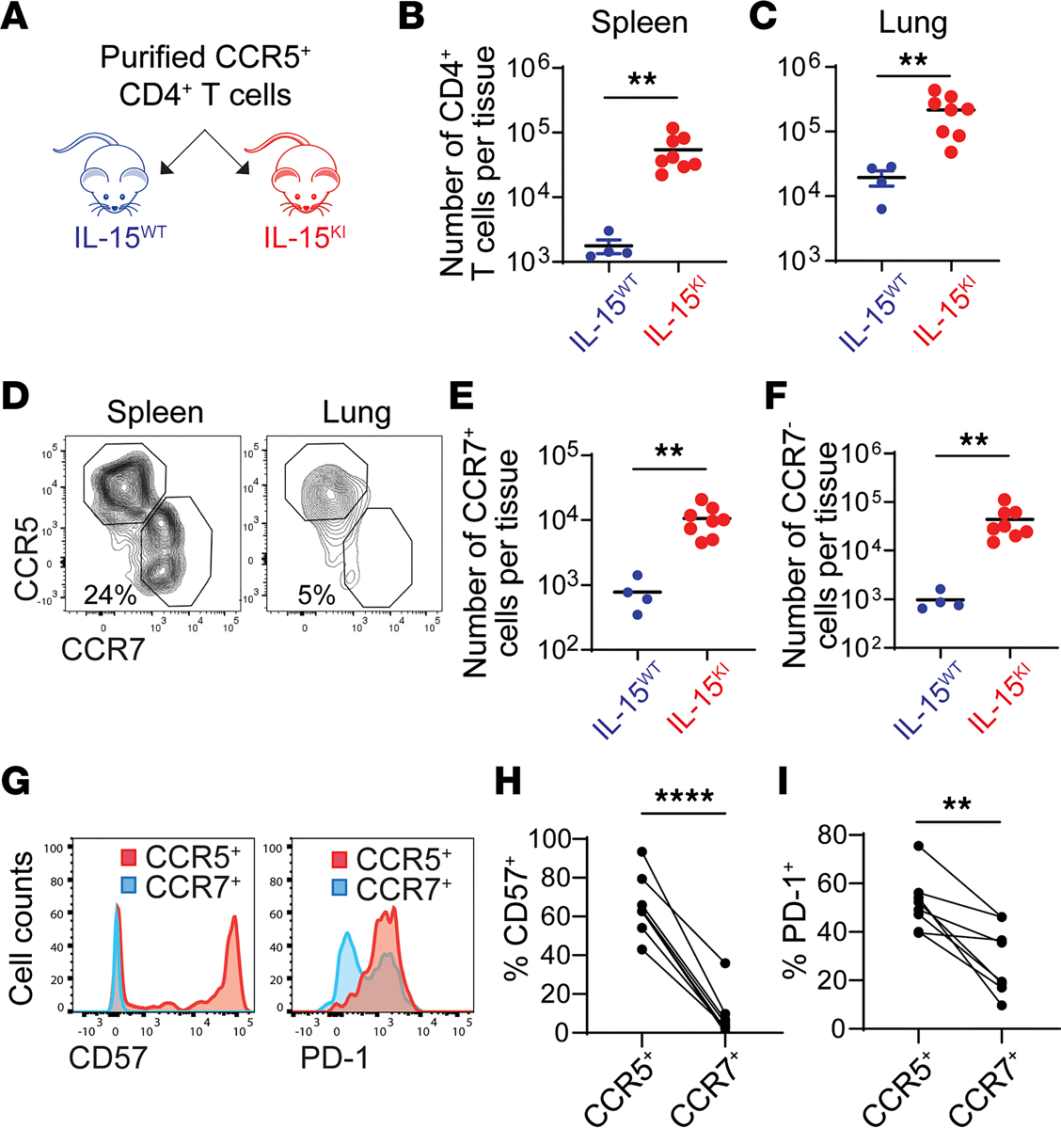
Survival and transdifferentiation of CCR5^+^CD4^+^ T cells in humanized mice. (**A**) Total blood CD4^+^ cells were costimulated with anti-CD3 and anti-CD28 antibodies for 3 days and then cultured in the presence of IL-2 for 6 days. CCR5^+^ cells were purified by sorting and were then transfused into 4 IL15^WT^ or 8 IL15^KI^ mice by retro-orbital injection. Each mouse received 3 × 10^6^ CCR5^+^ cells. Tissues were harvested on day 6 after transfusion. (**B** and **C**) The number of infused CD4^+^ T cells in spleens and lungs of IL15^WT^ and IL15^KI^ mice were determined by flow cytometry. (**D**–**I**) Transdifferentiation of CCR5^+^ cells into CCR7^+^ central memory cells in spleens and lungs of IL15^WT^ and IL15^KI^ mice. In **G**–**I**, the PD1 and CD57 expression analysis was only performed in IL15^KI^ mice. *n* = 8. *P* values were calculated using unpaired, 2-tailed *t* test (**B**, **C**, **E**, and **F**) or paired, 2-tailed *t* test (**H** and **I**). **P < 0.01; ****P < 0.0001.

Although transfusion of total CD4^+^ T cells clearly showed the impact of IL-15 on CCR5^+^CD4^+^ T cell differentiation, its effect on already differentiated CCR5^+^CD4^+^ T cells remained unclear. To further evaluate whether IL-15 enhanced survival of CCR5^+^CD4^+^ T cells in vivo, we purified CCR5^+^ cells and performed transfusion experiments (Figure 6A). The number of remaining infused cells was 5- to 10-fold greater in IL15^KI^ mice than in IL15^WT^ mice (Figure 6, B and C). In immunodeficient mice, the spleen is the only secondary lymphoid organ. Interestingly, a fraction of infused CCR5^+^CD4^+^ T cells turned off CCR5 expression and acquired a central memory phenotype (CCR7^+^), which likely drove their accumulation in spleens (Figure 6, D–F). These central memory–like cells had reduced levels of expression of T cell exhaustion markers, including PD-1 and CD57, when compared with those maintaining CCR5 expression (Figure 6, G–I).

**Figure 7:**
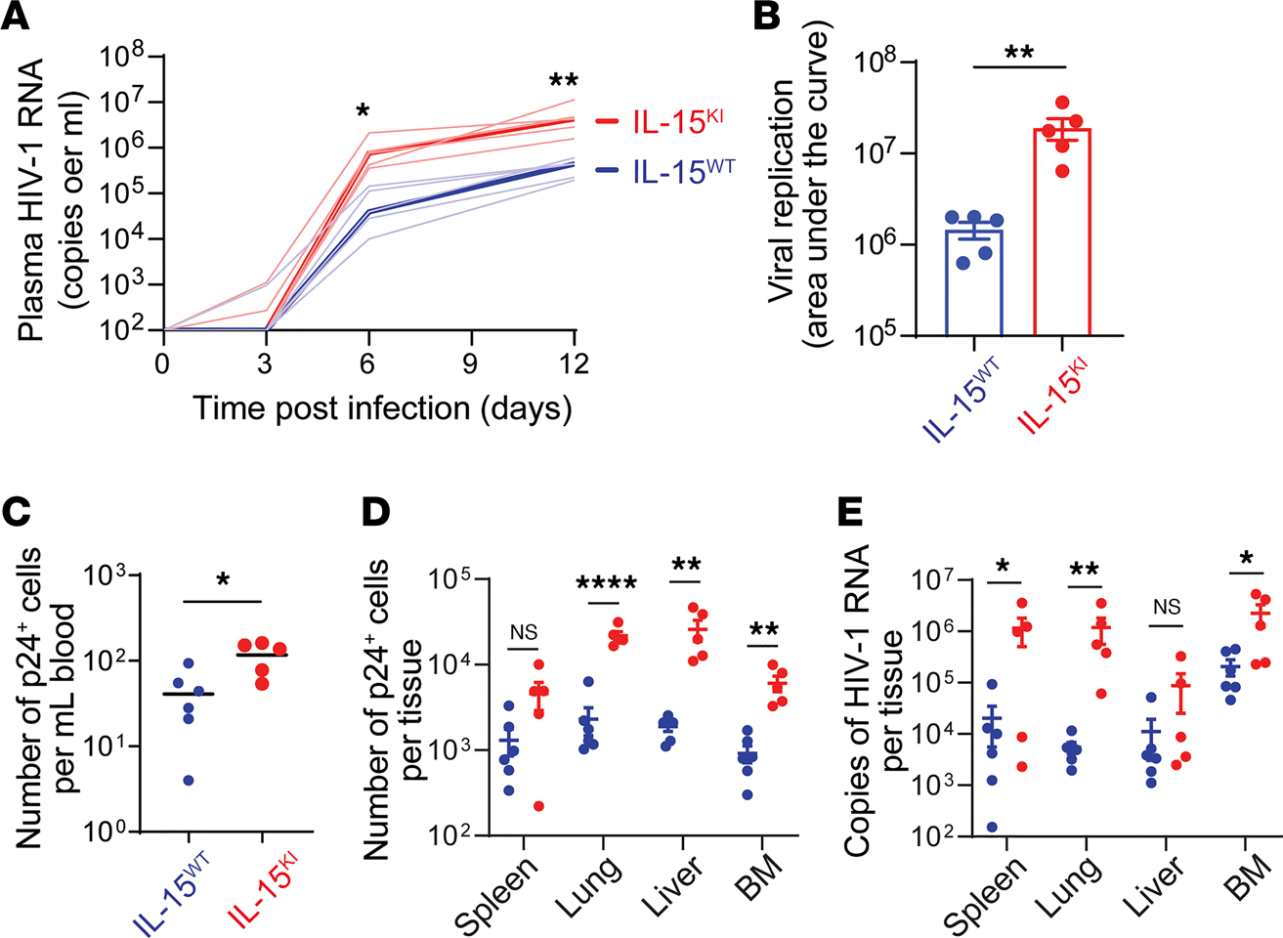
IL-15 promotes replication of CCR5-tropic HIV-1 in humanized mice. Total CD4^+^ cells from blood were costimulated with anti-CD3 and anti-CD28 antibodies for 3 days before transfused into IL15^WT^ and IL15^KI^ mice by retro-orbital injection. Each mouse received 5 × 10^6^ cells. Mice were infected with HIV_Ba-L_ 1 day after transfusion. (**A**) Plasma HIV-1 RNA was measured on 3, 6, and day 12 days after infection. (**B**) Plasma viral load AUC in **A** was calculated. In **A** and **B**, each group contained 5 mice. (**C**–**E**) Blood and tissues were collected on day 7 after infection. (**C** and **D**) Number of p24^+^ cells in blood and indicated tissues of IL15^WT^ and IL15^KI^ mice determined by flow cytometry. (**E**) Cell-associated HIV-1 RNA measured by RT-qPCR. In **C**–**E**, 5 IL15^WT^ and 6 IL15^KI^ mice were included. *P* values were calculated using 2-way ANOVA with Holm-Šidák multiple-comparison test (**A**, **D**, and **E**) or unpaired, 2-tailed *t* test (**B** and **C**). *P < 0.05; **P < 0.01; ****P < 0.0001.

Next, we tested the impact of IL-15 on R5-tropic HIV-1 infection in humanized mice. Plasma HIV-1 RNA became detectable in all mice on day 6 after infection and the viral loads in IL15^KI^ mice were 12- to 15-fold higher than in IL15^WT^ mice between day 6 and day 12 (Figure 7, A and B). The numbers of HIVp24^+^ cells in blood and all tissues of IL15^KI^ mice were 5- to 15-fold greater than their controls (Figure 7, C and D). The difference in HIVp24^+^ cells was not significant in the spleen, likely because the accumulation of central memory cells, which were no longer susceptible to R5-tropic viruses. Similarly, the levels of cell-associated HIV-1 RNA were approximately 100-fold higher in the tissues (except the liver) of IL15^KI^ mice (Figure 7E).

## Discussion

Due to its functions in the maturation and homeostasis of memory CD8^+^ T cells and NK cells (36), IL-15 is often considered a useful immunotherapeutic agent to treat HIV-1 infection. However, plasma IL-15 levels increase during HIV-1 infection (37) and are associated with high viral load or set point (27, 28, 38), likely because of the correlation between IL-15 and the frequency of HIV-1 target cells (25, 26, 29, 30). In the present study, we focused on the roles of IL-15 in the development of CCR5^+^CD4^+^ T cells, the major cellular targets of HIV-1. By studying human blood and lymphoid tissue CD4^+^ T cells in vitro and in humanized mice, we found that the vast majority of CCR5-expressing CD4^+^ T cells were short-lived CD127^–^ effector cells and produced high levels of IL-2Rβ and IL-2Rγ, which form the heterodimeric IL-15 receptor with intermediate affinity, sufficient to transduce downstream IL-15 signaling (39–41). Next, we found that IL-15 promoted the proliferation and survival of CCR5^+^CD4^+^ T cells purified from blood and lymphoid tissues. In addition, IL-15 treatment prolonged the lifespan of infected CCR5^+^CD4^+^ T cells and increased their virus production. By contrast, IL-7 treatment marginally expanded CCR5^+^CD4^+^ T cells in vitro, which suggests that IL-15 plays a more important role in the persistence of infected CCR5^+^CD4^+^ T cells. We further showed the expansion of CCR5^+^CD4^+^ T cells in humanized mice that produced physiologically relevant levels of human IL-15. Overall, our results demonstrate that IL-15 facilitates HIV-1 infection both in vitro and in humanized mice.

IL-15 plays confounding roles in HIV-1 infection. On the one hand, IL-15 augments anti–HIV-1 immune responses. On the other hand, it promotes HIV-1 infection by increasing the availability of CCR5^+^CD4^+^ T cells. For example, although blocking IL-15 signaling with neutralizing antibodies in RMs resulted in rapid depletion of NK cells and CD8^+^ effector memory T cells in blood and tissues, it did not alter the timing or magnitude of peak SIV infection (42, 43). Moreover, IL-15 treatment increased viral set point and accelerated disease progression despite enhancement of SIV-specific CD8^+^ T cell responses (44). These results and ours suggest that IL-15 treatment may drive high levels of HIV-1 replication, which cannot be fully counteracted by enhanced antiviral immune responses. Both IL-7 and IL-15 are implicated in the long-term maintenance and homeostatic proliferation of HIV-1 reservoirs in PLWH on ART (45, 46). Interestingly, pre-ART plasma IL-15 levels positively correlated with both pre- and on-ART frequency of HIV-1 DNA in all subsets of CD4^+^ T cells in all study participants (46), suggesting a central role of IL-15 in the initial seeding of HIV-1 reservoirs. As IL-15 improves the viability of HIV-1–infected CCR5^+^CD4^+^ T cells and may drive their expansion once the integrated provirus becomes transcriptionally silent, the IL15^KI^ and control mice under ART will be useful to evaluate the effect of IL-15 on the seeding and stability of HIV-1 reservoirs in vivo. In vivo studies will help understand how HIV-1–infected CCR5^+^CD4^+^ T cells undergo transcriptional and phenotypic changes to become either long-lived central memory cells or more active effector memory cells while carrying a latent provirus.

Since IL-15 can induce HIV-1 transcription in latently infected CD4^+^ T cells (47), the IL-15 receptor superagonist complex N-803 is considered a promising agent that leads to both viral latency reversal and enhanced immune clearance (48). However, CCR5^+^CD4^+^ T cells contribute a significant portion of the latent reservoir for HIV-1 in PLWH on ART (24). In addition, IL-15–expanded CCR5^+^CD4^+^ T cells coexpress PD-1, TIGIT, and LAG3 (Figure 3C and Supplemental Figure 2), which are highly enriched for transcription-competent viral reservoirs in PLWH (49, 50). In NHPs, N-803 alone did not reactivate latent SIV, while its administration in conjunction with CD8^+^ cell depletion led to virus reactivation (51). It is possible that CD8^+^ cell depletion made IL-15 more available to CD4^+^ T cells and drove expansion of CCR5^+^CD4^+^ T cells, which likely contributed to the reactivation of latent SIV. Therefore, in addition to latency reversal, studies on the potential effects of IL-15 signaling on expansion of the viral reservoir in CCR5^+^CD4^+^ T cells are warranted.

## Methods

### Human samples.

Anonymous peripheral blood samples were acquired from the Mississippi Valley Regional Blood Center as waste cellular products. Human tonsils were collected from elective tonsillectomies from Children’s Hospital in St. Louis, which were provided as surgical waste, with no identifiers attached. De-identified lymph node samples were obtained from fine needle aspiration. Mononuclear cells were isolated using Ficoll-Paque PLUS (GE Healthcare) by density gradient separation following the manufacturer’s protocol. CD4^+^ cell negative selection (MojoSort Human CD4 T Cell Isolation Kit, BioLegend) was performed and CD4^+^ T cells were sorted using a FACSAria II sorter (BD Bioscience) based on the expression of the following markers: CD3 (APC-Cy7), CD4 (APC), CD45RO (PE), CCR5 (BV421), and CCR7 (FITC).

### Plasmids and viruses.

The plasmid pNL4-3Δenv-EGFP (ARP-11100) and the HIV_Ba-L_ viruses (ARP-510) were obtained from the NIH HIV reagent program. The CCR5-tropic HIV-1 reporter viruses were produced by transient transfection of HEK293T cells with pNL4-3Δenv-EGFP and the expression plasmid for the Yu2 envelope. HIV_Ba-L_ was propagated in PHA-stimulated PBMCs. A Lenti-X Concentrator (Takara) was used to generate concentrated viral stocks. Viral titers were determined by p24 ELISA (XpressBio). For in vitro infection, spin inoculation at 1,200*g* for 2 hours was performed at a dose of 25 ng p24 per 10^6^ CD4^+^ T cells for HIV_Ba-L_, or 70 ng p24 per 10^6^ CD4^+^ T cells for the reporter virus NL4-3Δenv-EGFP. For in vivo infection with HIV_Ba-L_, 20 ng p24 in 100 μL PBS per mouse was used via retro-orbital injection.

### Mice.

The generation of mice with human *IL15* knockin on a 129 × BALB/c (N3) genetic background was performed using VelociGene technology by Regeneron Pharmaceuticals. Mice were bred to a *Rag2^−/−^ IL2rg^−/−^* background with homozygous *IL15* knockin. IL15^KI^ and IL15^WT^ mice were houses in specific pathogen–free animal facilities with appropriate biosafety containment at Washington University in St. Louis. Both male and female mice were used between the ages of 6 and 10 weeks.

### Isolation and culture of CD4^+^ T cells.

CD4^+^ T cells were purified by negative selection with a MojoSort Human CD4 T Cell Isolation Kit (BioLegend) or EasySep Human CD4^+^ T Cell Isolation Kit (STEMCELL Technologies). Purified CD4^+^ T cells (1 × 10^6^ cells/mL) were cultured in RPMI 1640 medium supplemented with 10% FCS, penicillin (100 U/mL), and streptomycin (100 μg/mL). To generate activated cells, CD4^+^ T cells were stimulated with plate-bound anti-CD3 and anti-CD28 antibodies at 1 μg/mL (BioLegend) for 3 days in the presence of 20 ng/mL IL-2 (BioLegend). Stimulated CD4^+^ T cells were cultured with 20 ng/mL IL-7 (BioLegend) or IL-15 (BioLegend). Fresh medium was provided every 3 days to maintain the cell densities at 1 × 10^6^ to 2 × 10^6^ cells/mL.

### Mass cytometry.

For mass cytometry analysis, metal-tagged antibodies were purchased from Fluidigm or custom-conjugated using the Maxpar X8 Antibody Labeling Kit according to the manufacturer’s instructions (Fluidigm). All metal-conjugated antibodies used in this study are included in Supplemental Table 1. Unstimulated or activated CD4^+^ T cells (5 × 10^6^) were washed and resuspended in CyFACS buffer (0.1% BSA, 0.02% NaN_2_, 2 mM EDTA in CyPBS, Rockland). Human Fc-receptor blocking solution (Affymetrix) was added to each sample for 10 minutes at room temperature. Cells were then stained with surface antibodies for 60 minutes on ice. Surface antibodies were washed away using CyPBS. Subsequently, cells were exposed to 2.5 μM cisplatin (Enzo Life Sciences) as a viability indicator before being fixed in 4% paraformaldehyde. Fixed cells were barcoded with a Fluidigm Cell-ID 20-Plex Pd Barcoding Kit. All mass cytometry data were collected on a CyTOF2 mass cytometer (Fluidigm) and analyzed using Cytobank. Cell gating was performed as described in Bandyopadhyay et al. (52) and gated based on EQ1^–^, DNA^+^, singlet^lo^, cisplatin^–^, CD45^+^, CD3^+^, and CD4^+^. viSNE analysis, which uses Cytobank applications employing the Barnes-Hut implementation of the t-stochastic neighbor embedding (t-SNE) algorithm, was performed as previously described (53).

### Flow cytometry.

Cell viability was determined by the APC Annexin V Apoptosis Detection Kit with 7-AAD (BioLegend). For HIV-1–infected cells, cells were fixed using 4% paraformaldehyde for 10 minutes after antibody staining. The Cytofix/Cytoperm kit (BD Biosciences) was used for intracellular p24 staining. To track cell proliferation in vitro and in mice, activated CD4^+^ T cells were labeled with the CellTrace CFSE or Violet at 5 μM (Thermo Fisher Scientific). To label dead cells, 7-AAD was used for uninfected cells and the Zombie Aqua Fixable Viability Kit (BioLegend) was used for infected cells prior to fixation. All flow cytometry data were acquired by BD LSR Fortessa, BD X20, or BD Accuri C6 cytometer, and FlowJo software was used for data analysis. Sorting of CCR5^+^ and CCR5^–^ CD4^+^ T cells and HIV-1–infected GFP^+^ cells was performed using a BD FACSAria II flow cytometer. The following antibodies used for surface and intracellular staining were purchased from BioLegend: mCD45 (clone 30-F11), hCD45 (clone HI30), hCD3 (clone HIT3a), hCD4 (clone OKT4), hCCR5 (clone J418F1), hCCR7 (clone G043H7), hCD45RO (clone UCHL1), hCD45RA (clone HI100), hCD57 (clone HNK-1), hPD1 (clone EH12.2H7), hCD122 (clone TU27), and hCD132 (clone TUGh4). Anti–HIV-1 p24 (clone KC57-RD1) was from Beckman Coulter and anti-hCD215 (clone JM7A4) was from R&D Systems.

### RNA-Seq.

CCR5^+^ and CCR5^–^ CD4^+^ T cells were sorted, and total RNA was extracted using a Direct-zol RNA kit (Zymo Research). The cDNA library was prepared using strand-specific RNA-Seq protocols. Samples were prepared according to the library kit manufacturer’s protocol, indexed, pooled, and sequenced on an Illumina HiSeq. Basecalls and demultiplexing were performed with Illumina’s bcl2fastq software and a custom python demultiplexing program with a maximum of 1 mismatch in the indexing read. RNA-Seq reads were then aligned to the Ensembl release 76 primary assembly with STAR version 2.5.1a (54). Gene counts were derived from the number of uniquely aligned unambiguous reads by Subread:featureCount version 1.4.6-p5 (55). Sequencing performance was assessed for the total number of aligned reads, total number of uniquely aligned reads, and features detected. The ribosomal fraction, known junction saturation, and read distribution over known gene models were quantified with RSeQC version 2.6.2 (56). Counts were first filtered by removing genes where greater than half of the samples had counts per million (cpm) values less than 1 using Python version 3.8.5 (https://www.python.org/). Differential expression analysis was assessed using the R/Bioconductor package DESeq2 (57). Volcano plot of differentially expressed genes was created using the R/Bioconductor package EnhancedVolcano (58). For GSEA, genes were preranked using log_2_(fold change) and analysis was done via MSigDB’s GSEA desktop software using the Immunologic Gene Signature gene sets (59, 60). Barcode plots were generated using the R/Bioconductor package fGSEA. Fragments per kilobase of exon per million reads mapped (FPKMs) of DESeq2-normalized counts were used for calculating *z* scores in Microsoft Excel for generating heatmaps of the top genes from the selected gene sets. RNA-Seq data were submitted to the NCBI Gene Expression Omnibus GEO repository (GEO GSE199729).

### Animal experiments.

IL15^WT^ and IL15^KI^ mice were used for transfusion of human blood CD4^+^ T cells by retro-orbital injection. CD4^+^ T cells were stimulated with anti-CD3 and anti-CD28 antibodies for 3 days. For transfusion of total CD4^+^ T cells, each mouse received 5 × 10^6^ activated cells. For transfusion of CCR5^+^CD4^+^ T cells, cell sorting was performed, and each mice received 3 × 10^6^ purified CCR5^+^ cells. To track cell proliferation in vivo, activated CD4^+^ T cells were labeled with CSFE and infused into IL15^WT^ and IL15^KI^ mice (8 × 10^6^ cells/mouse). For in vivo HIV-1 infection, mice received total CD4^+^ T cells (5 × 10^6^ cells per mouse) 24 hours before infection with HIV_Ba-L_ at a dose of 20 ng p24.

### ELISA.

IL15^WT^ and IL15^KI^ mice were injected with lipopolysaccharide (Sigma-Aldrich) at a dose of 0.4 mg/kg. Human IL-15 protein concentration was measured in the plasma 6 hours later using a human IL-15 Quantikine ELISA kit (R&D Systems).

### Viral quantification and gene expression analysis.

Supernatant HIV-1 particles were quantified by p24 ELISA (XpressBio). For quantification of cell-free HIV-1 RNA, a Quick-RNA Viral Kit (Zymo Research) was used to extract viral RNA from culture supernatant or peripheral blood. For quantification of cell-associated HIV-1 RNA, a Directzol RNAmini Plus kit (Zymo Research) was used to extract total RNA from cell cultures and mouse tissues. Extracted RNA was reverse transcribed with SuperScript III Reverse Transcriptase and random primers (Invitrogen), and cDNA was used for real-time PCR. The primers and probe used for HIV-1 *gag* mRNA measurement were described previously (61). The detection limit for HIV-1 RNA was 200 copies/mL.

To determine host gene transcription levels, RNA polymerase II subunit A (*POLR2A*) was used as a reference gene. Predesigned gene-specific TaqMan assays were purchased from Thermo Fisher Scientific for quantitative PCR. The TaqMan assay IDs are listed in Supplemental Table 1.

### Statistics.

Statistical analyses were performed using Prism software (GraphPad Software). A *P* value of less than 0.05 was considered significant. Statistical tests are listed in the figure legends.

### Study approval.

All animal experiments were approved by the Institutional Animal Care and Use Committee of Washington University School of Medicine.

## Author contributions

LS, YL, and HG designed the experiments and performed analyses. YL and HG performed experiments. KMC performed the RNA-Seq analyses. LS wrote the manuscript.

## Supplementary Material

Supplemental data

## Version 1 02/23/2023

In-Press Preview

## Version 2 04/10/2023

Electronic publication
