# Supplementary material for: IL-15 enhances HIV-1 infection by promoting survival and proliferation of CCR5+CD4+ T cells
Source: JCI Insight. 2023 Apr 10;8(7):e166292. doi: 10.1172/jci.insight.166292 (PMC10132148; doi:10.1172/jci.insight.166292)
Supplement: Supplemental data [file jciinsight-8-166292-s135.pdf]

Fig. S1

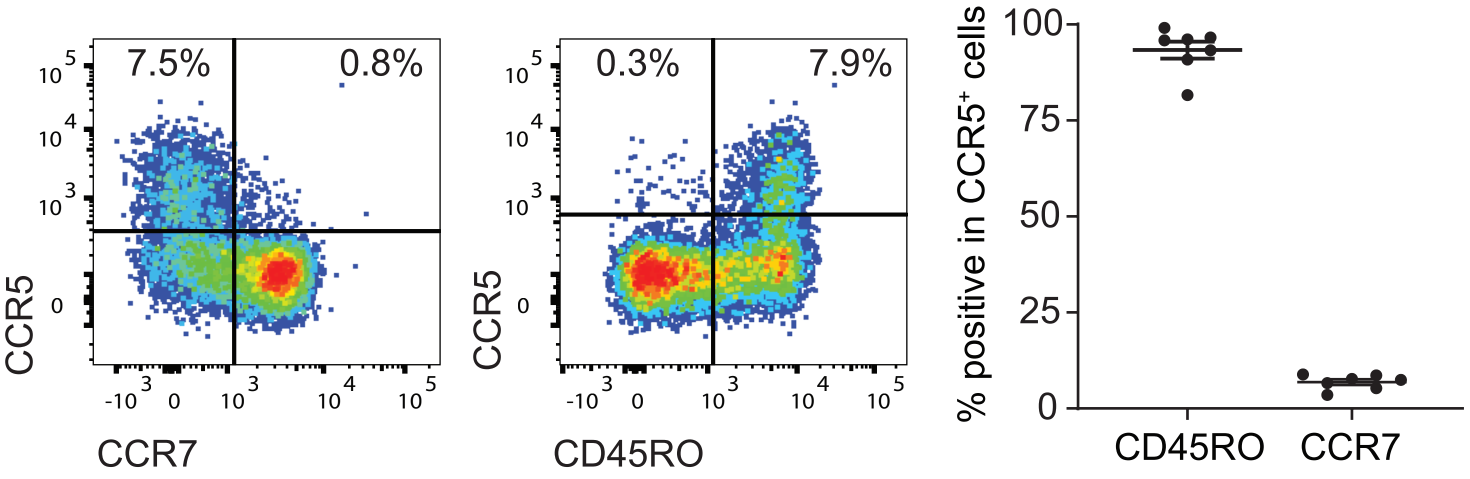

7.5%
0.8%
CCR5
CCR7
0.3%
7.9%
CCR5
CD45RO
% positive in CCR5+ cells
100
75
50
25
0
CD45RO
CCR7

Supplemental Figure 1. $CCR5^+CD4^+$ T cells are effector or effector memory cells.

Expression of CD45RO and CCR7 in unstimulated $CCR5^+$ and $CCR5^-CD4^+$ T cells. $CD4^+$ T cells from 7 healthy blood donors were included.

Fig. S2

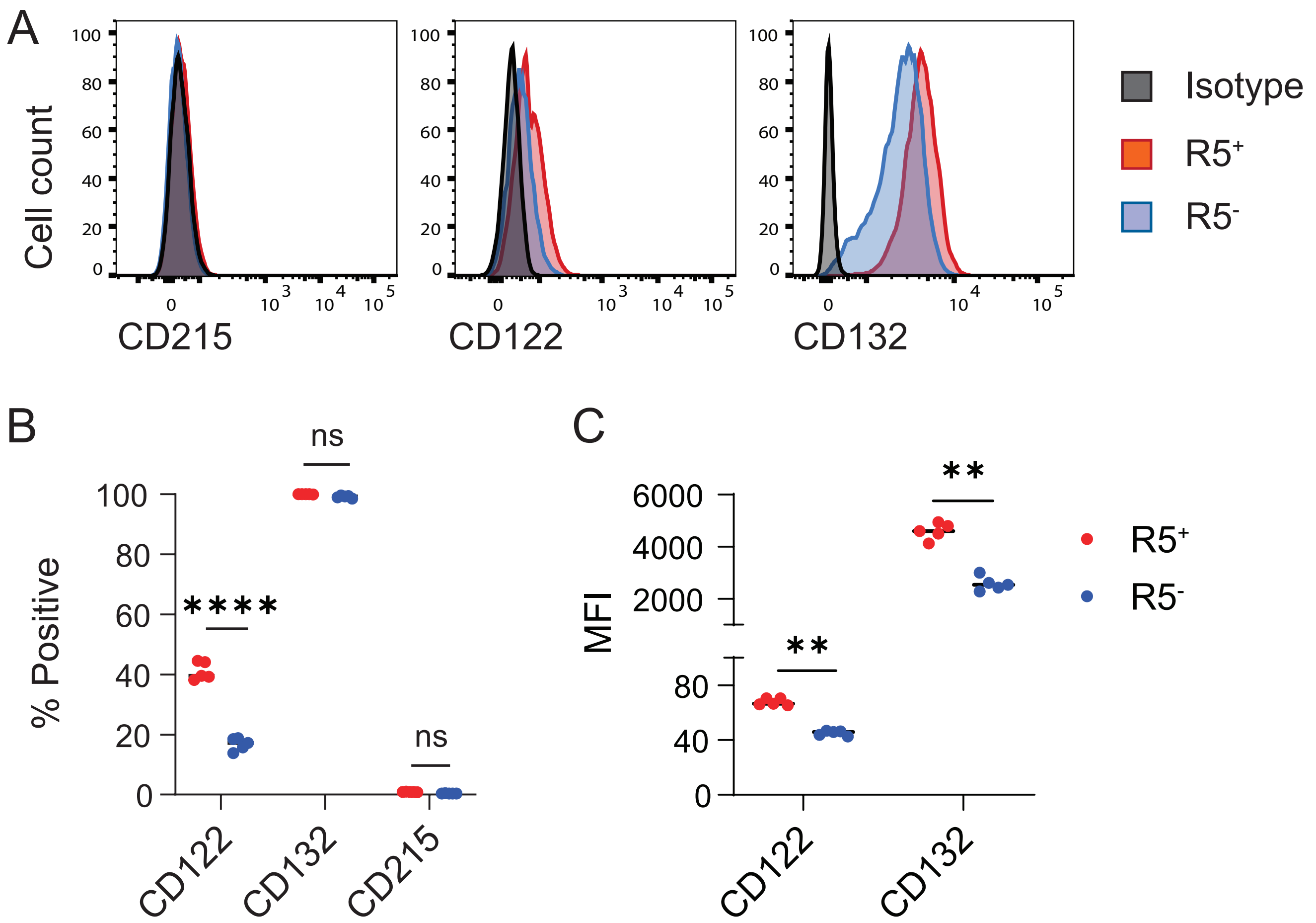
A
Cell count
CD215
CD122
CD132
Isotype
R5+
R5-
B
% Positive
ns
****
ns
CD122
CD132
CD215
C
MFI
**
**
CD122
CD132
R5+
R5-

Supplemental Figure 2. Expression of IL15 receptor subunits.

(**A-C**) Total $CD4^+$ T cells isolated from peripheral blood of five healthy donors were stimulated with anti-CD3 and anti-CD28 antibodies for 3 days. Activated cells were cultured for 3 additional days without any cytokine before flow cytometry analysis. *p* values were calculated using two-way ANOVA and Holm-Sidak multiple comparisons test. *** $p < 0.001$; **** $p < 0.0001$.

Fig. S3

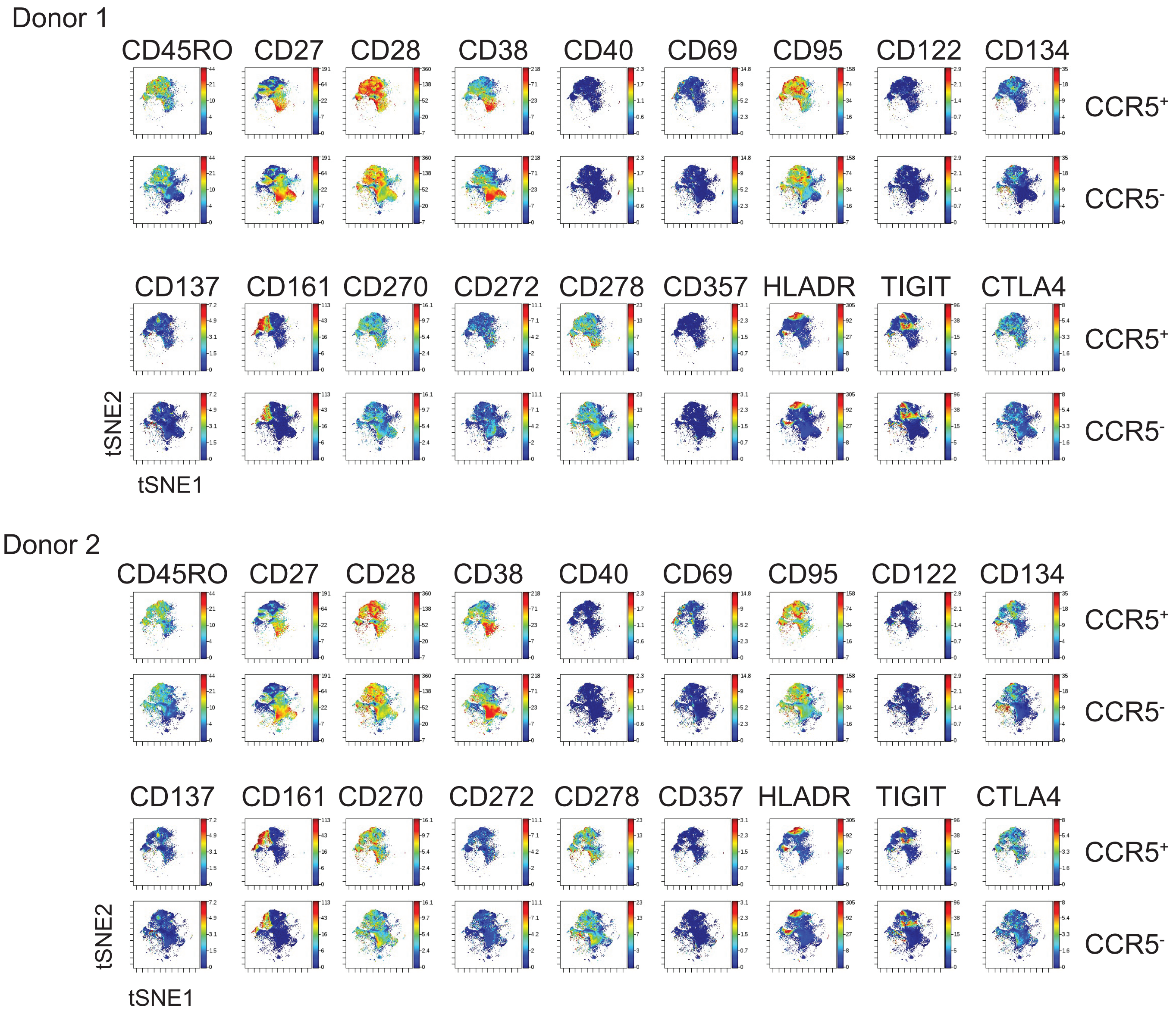

Donor 1
CD45RO CD27 CD28 CD38 CD40 CD69 CD95 CD122 CD134
CCR5+
CCR5-
CD137 CD161 CD270 CD272 CD278 CD357 HLADR TIGIT CTLA4
CCR5+
CCR5-
tSNE2
tSNE1
Donor 2
CD45RO CD27 CD28 CD38 CD40 CD69 CD95 CD122 CD134
CCR5+
CCR5-
CD137 CD161 CD270 CD272 CD278 CD357 HLADR TIGIT CTLA4
CCR5+
CCR5-
tSNE2
tSNE1

Supplemental Figure 3. CyTOF analysis of $CD4^+$ T cells expanded by IL15.

Total $CD4^+$ T cells isolated from peripheral blood of two healthy donors were stimulated with anti-CD3 and anti-CD28 antibodies for 3 days. Activated cells were incubated with IL15 for 6 days before CyTOF analysis. Live $CD45^+$ singlets were exported for analysis by visualization of stochastic neighbor embedding (viSNE) analysis methods. The viSNE plots illustrate the expression intensity of indicated genes in $CCR5^+$ or $CCR5^-CD4^+$ T cells. The coloration is proportional to the expression intensity (blue = low, red = high).

Fig. S4

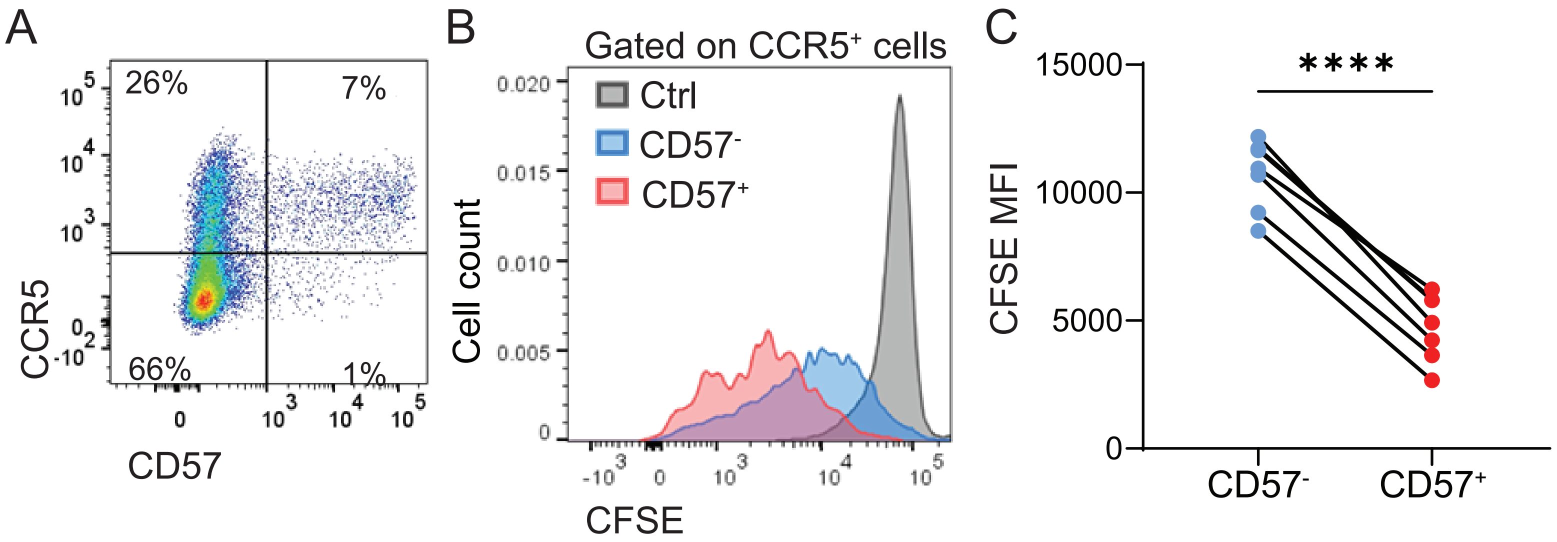

A
26%
7%
66%
1%
CCR5
CD57
B
Gated on CCR5⁺ cells
Ctrl
CD57⁻
CD57⁺
Cell count
0.020
0.015
0.010
0.005
0
CFSE
C
****
CFSE MFI
15000
10000
5000
0
CD57⁻
CD57⁺

Supplemental Figure 4. Proliferation of the $CD57^+$ subset of $CCR5^+CD4^+$ T cells driven by IL15. Total $CD4^+$ T cells isolated from peripheral blood of seven healthy donors were co-stimulated with anti-CD3 and anti-CD28 antibodies for 3 days. Activated cells were stained with CFSE and incubated with IL15 for 6 days before flow cytometry analysis. (**A**) Most CD57-expressing cells are $CCR5^+$. (**B-C**) IL15 expands the $CD57^+$ subset of $CCR5^+$ cells. *p* values were calculated using paired t test. **** $p < 0.0001$.

Fig. S5

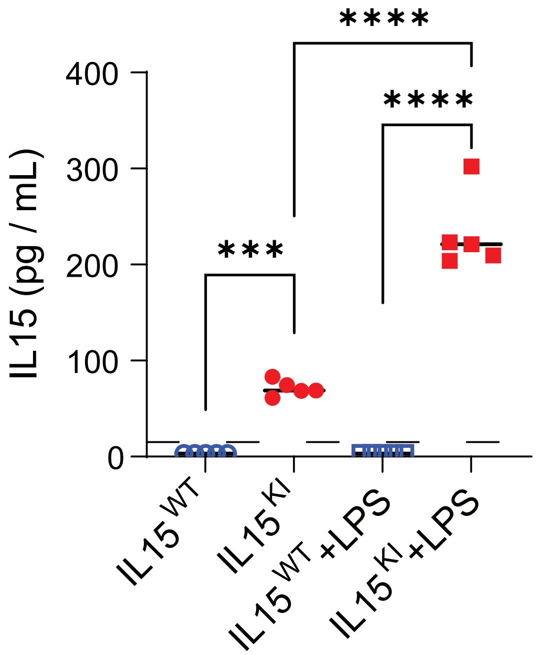
****
****
***
400
300
200
100
0
IL15 (pg / mL)
IL15 WT
IL15 KI
IL15 WT +LPS
IL15 KI +LPS

Supplemental Figure 5. Human IL15 concentration in $IL15^{KI}$ mice. Human IL15 measured in the plasma of $IL15^{KI}$ and $IL15^{WT}$ mice following LPS treatment (0.4mg/kg). *p* values were calculated using one-way ANOVA with Tukey test, ***$p < 0.001$, ****$p < 0.0001$. Detection limit is 3.91pg /mL. Open symbols represent no detection.

Fig. S6

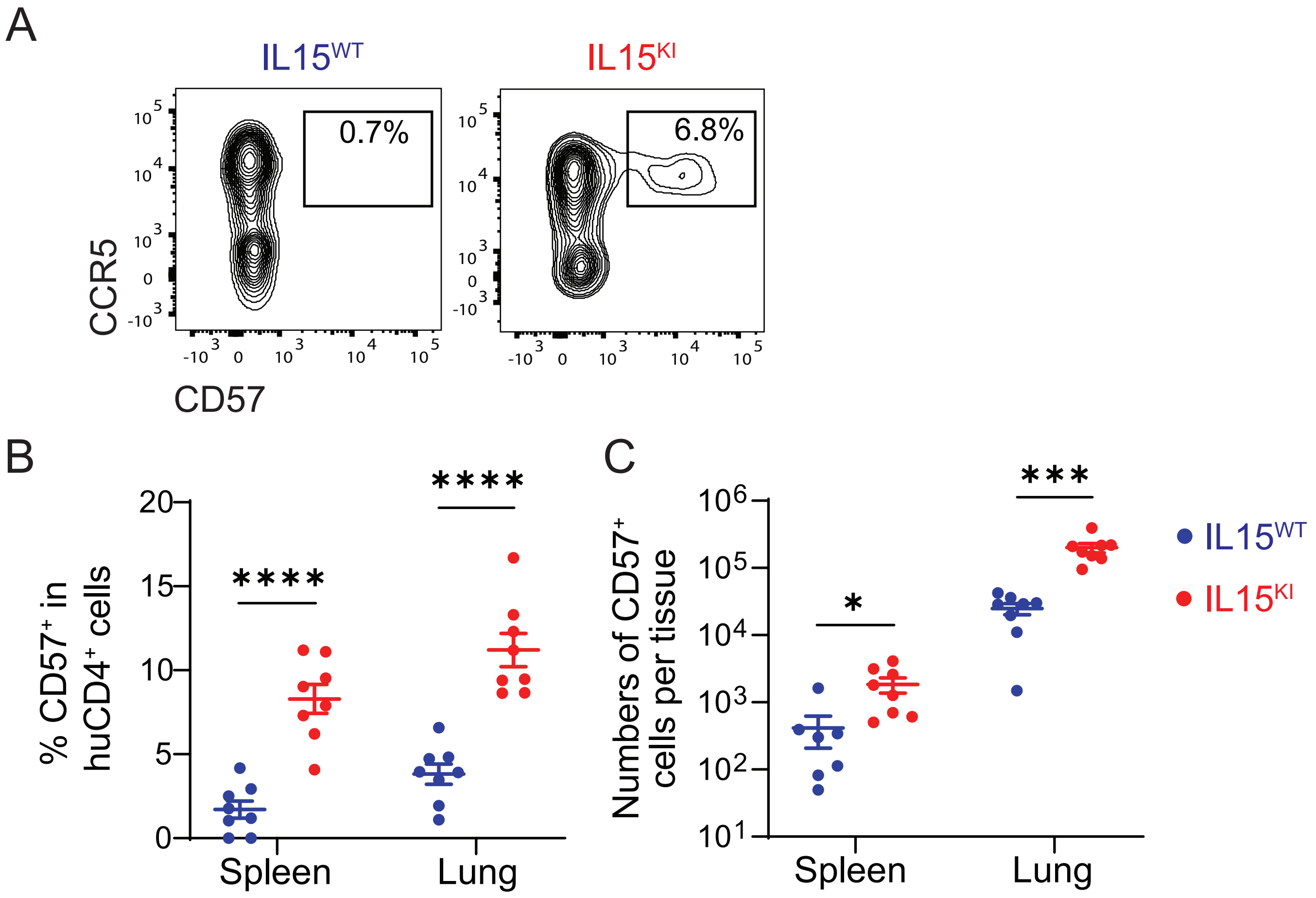

A
IL15WT
IL15KI
0.7%
6.8%
CCR5
CD57
B
% CD57+ in huCD4+ cells
****
****
Spleen
Lung
C
Numbers of CD57+ cells per tissue
*
***
Spleen
Lung
IL15WT
IL15KI

Supplemental Figure 6. Expansion of the CD57^{+} subset of CCR5^{+}CD4^{+} T cells in IL15^{KI} mice. Total CD4^{+} cells from blood were co-stimulated with anti-CD3 and anti-CD28 antibodies for 3 days before transfused into IL15^{WT} and IL15^{KI} mice by retro-orbital injection. Each mouse received $5*10^{6}$ cells. Blood and tissues were collected on day 6 post transfusion for flow cytometry analysis. (**A**) Expression of CD57 in CD4^{+} T cells in lungs of IL15^{WT} and IL15^{KI} mice. (**B-C**) The percentage (B) and number (C) of human CD57^{+} cells in spleens and lungs from IL15^{WT} and IL15^{KI} mice. p values were calculated using two-way ANOVA and Holm-Sidak multiple comparisons test. * $p < 0.05$; *** $p < 0.001$; **** $p < 0.0001$.
